# Growth hormone exacerbates diabetic renal damage in male but not female rats

**DOI:** 10.1186/2042-6410-4-12

**Published:** 2013-06-27

**Authors:** Jennifer L Whitney, Christine Maric Bilkan, Kathryn Sandberg, Adam K Myers, Susan E Mulroney

**Affiliations:** 1 Department of Physiology and Biophysics, Georgetown University Medical Center, Washington, DC 20057-1640, USA; 2 Department of Physiology and Biophysics, University of Mississippi Medical Center, Jackson, MS, USA; 3 Department of Medicine, Georgetown University Medical Center, Washington, DC, USA; 4 Center for the Study of Sex Differences in Health, Aging and Disease, Georgetown University Medical Center, Washington, DC, USA

**Keywords:** Sex Differences, Gender, Glomerulosclerosis, Renal Injury, TGF-β, Inflammation

## Abstract

**Background:**

Human and animal studies support the idea that there are sex differences in the development of diabetic renal disease. Our lab and others have determined that in addition to Ang II (through the AT_1_R), growth hormone (GH) contributes to renal damage in models of renal failure; however, the impact of sex and GH on the mechanisms initiating diabetic renal disease is not known. This study examined the effect of sex and GH on parameters of renal damage in early, uncontrolled streptozotocin (STZ)-induced diabetes.

**Methods:**

Adult male and female Sprague–Dawley rats were injected with vehicle (control), STZ, or STZ + GH and euthanized after 8 weeks.

**Results:**

Mild but significant glomerulosclerosis (GS) and tubulointerstitial fibrosis (TIF) was observed in both kidneys from male and female diabetic rats, with GH significantly increasing GS and TIF by 30% and 25% in male rats, *but not in female rats*. STZ increased TGF-β expression in both kidneys from male and female rats; however, while GH had no further effect on TGF-β protein in diabetic females, GH increased TGF-β protein in the male rat’s kidneys by an additional 30%. This sex-specific increase in renal injury following GH treatment was marked by increased MCP-1 and CD-68+ cell density. STZ also reduced renal MMP-2 and MMP-9 protein expression in both kidneys from male and female rats, but additional decreases were only observed in GH-treated diabetic male rats. The sex differences were independent of AT_1_R activity.

**Conclusions:**

These studies indicate that GH affects renal injury in diabetes in a sex-specific manner and is associated with an increase in pro-inflammatory mediators.

## Background

Growth hormone (GH) has been shown to exacerbate progression of kidney damage in a number of experimental models of renal disease [1-4], including diabetes [5]. Indeed, the kidney appears to be very sensitive to GH, as seen in male transgenic mice overexpressing GH that present with glomerular hypertrophy, increased glomerular cell turnover, and increased extracellular matrix accumulation leading to diffuse glomerulosclerosis [6,7]. It has also been reported that diabetic patients present with dysregulation of GH, which ultimately results in overall GH hypersecretion [8]. Diabetic mice treated with a specific GH receptor antagonist showed none of the diabetes-associated renal hypertrophy or glomerular enlargement and had lower urinary albumin excretion than untreated diabetic mice [5,9]. These results were confirmed in GH receptor antagonist transgenic mice that were protected against diabetes-induced renal changes [10]. Collectively, these findings strongly suggest that GH plays a role in the development of diabetic renal disease; however, the mechanism whereby GH acts on the kidney is not completely understood. While GH is known to stimulate liver and tissue insulin-like growth factor (IGF-1), this IGF-I associated mechanism does not appear to play a role in diabetic renal damage [8].

In the streptozotocin (STZ)-induced diabetic rat, a common diabetic rat model, normal GH secretion is dysregulated [11]. Indeed, by 6 h post-STZ, pulsatile GH secretion is dramatically reduced in male rats and is abolished by 12 h [11]. Suppression of plasma GH by STZ is also observed in female rats (unpublished data from this laboratory). This decrease seen in GH levels is opposite of what is seen in patients with type 1 diabetes, where GH levels are dysregulated, and usually elevated compared to nondiabetic control patients [8,12]. We hypothesize that the loss of GH in the STZ model impacts the development of diabetic kidney disease and that using an STZ model with GH supplementation will more accurately reflect the human condition.

Another important aspect of human diabetes is the increasing evidence of sex differences in the development and/or severity of diabetic renal disease [13-16], and although sex hormones appear to be key factors [17,18], the exact reasons for the differences are still not clear. In addition, both sex and diabetes are key factors contributing to cardiovascular diseases [19-22]. Our lab has previously reported the link between GH and the development of renal disease following unilateral nephrectomy [4,17]. More importantly, we have identified sex differences in the effects of GH on remnant kidney growth and pathology and determined that GH is crucial in compensatory renal growth in male animals, but not in female animals [17,23]. While many renal diseases have been shown to progress at different rates between the sexes, these findings indicate that there are differential pathologic pathways governed by gonadal steroids, with testosterone promoting a GH-dependent, hypertrophic renal growth, and 17β-estradiol promoting IGF-1-associated hyperplastic renal growth [16,17,23]. On this basis, we hypothesize that there also may be sex differences in GH-mediated diabetic renal damage.

Taken together, there is a strong rationale for sex differences in the development of diabetic renal disease, and GH may be a contributing factor to these differences. Since sex hormones can affect renal hemodynamics, mesangial cell proliferation, extracellular matrix metabolism, as well as synthesis and release of vasoactive agents [15], their role in diabetes could help define sex-specific therapeutic interventions. Thus, the objective of this study was to determine the effects of sex and GH on mediators of renal damage and subsequent renal disease in the initial stage of uncontrolled diabetes.

## Methods

### Animals

Male and female Sprague–Dawley rats (Harlan, Madison, WI, USA) (12–14 weeks of age), were randomly divided into three treatment groups for each sex: control (*n* = 10), streptozotocin (*n* = 8), and STZ + GH (*n* = 9). After fasting animals overnight, diabetes was induced by an intraperitoneal injection of STZ (55 mg/kg in 100 μl 0.1 M citrate) in the STZ and STZ + GH groups, whereas control animals were injected with vehicle (100 μl 0.1 M citrate). Because there are sex differences in GH secretory patterns (regular high pulses in males [24]) and higher baseline, erratic, and low pulses in females [23], we performed a pilot study using twice daily GH injections (which we have used successfully in many studies) vs continual GH release via an osmotic minipump, and the damage to both male and female kidneys were similar between each method (data not shown). We decided to use the GH injections primarily because of our past success with this method. The GH-supplemented diabetic rats (STZ + GH) were injected subcutaneously with GH twice daily over the 8-week duration of the experiment (2.5 μg in 100 μl saline between 9–11 a.m. and 3–5 p.m.), whereas the non-GH-treated rats were injected twice daily with vehicle (100 μl saline). This dose has been shown to increase plasma GH to 80–100 mmol/l within 30 min, which is similar to levels observed in male rats [17]. All animals were fed a phytoestrogen-free diet (Harlan) and given tap water *ad libitum*. Twenty-four-hour urine was collected from rats placed in metabolic cages after 0, 4, and 8 wks of treatment. Diabetic animals were not treated with insulin in order to mimic the changes present in early uncontrolled diabetes. Plasma glucose was determined using a One-Touch Ultra glucometer (LifeScan, Milpitas, CA, USA) from blood collected via tail puncture at day 0 and thereafter on a weekly basis during the course of the entire experiment. Animals were sacrificed by decapitation. The left kidney was weighed, removed, and snap-frozen in liquid nitrogen for analysis of TGF-β, MMP-2, MMP-9 protein expression by Western blotting and AT_1_R activity by radioligand binding. The right kidney was fixed in 4% paraformaldehyde for assessment of glomerulosclerosis and tubulointerstitial fibrosis as well as tissue expression of monocyte chemoattractant protein-1 (MCP-1)- and CD68-positive cells by immunohistochemistry. Urine albumin concentration was determined by ELISA (Exocell, Philadelphia, PA, USA) and multiplied by urine volume to calculate urine albumin excretion. All procedures were approved by the Georgetown University Animal Care and Use Committee.

### Western blotting

Renal cortex was prepared for Western blotting by rapidly dissecting frozen kidney tissue on ice, followed by homogenization using the Bio-Rad’s Criterion system (Hercules, CA, USA), as previously described [25]. Briefly, total protein (30 mg) was loaded in each lane followed by electrophoresis on 10% polyacrylamide gels. The gels were incubated with one of the following primary antibodies: TGF-β (1:1,000, Santa Cruz Biotechnology, Santa Cruz, CA, USA), MMP-2 (1:500, Oncogene Science, Cambridge, MA, USA), and MMP-9 (1:500, Oncogene Sciences). Specific bands were detected by chemiluminescent peroxidase (Amersham, Buckinghamshire, UK) and recorded on X-ray film. Relative density of bands was determined by normalizing each band to lane total protein density determined by a Coomassie blue stain. Corresponding samples of male and female homogenates were compared side by side on the same gel.

### AT_1_R binding

Because we have previously reported sex-dependent increases in AT_1_R binding by GH in another model of renal disease [17] and identified sex-hormone-dependent effects on AT_1_R binding [25], we examined whether renal AT_1_R binding was altered by GH in diabetic male and female animals. Specific AT_1_R binding was determined in isolated glomeruli (5 μg/tube) using ^125^I-[Sar^1^, Ile^8^]Ang II as the radioligand, as previously described [22]. Specific binding was defined as the total binding in the presence of radioligand minus the amount of binding in the presence of radioligand and 500 μM cold [Sar^1^, Ala^8^]Ang II.

### Glomerulosclerotic index

Periodic acid Schiff-stained, paraffin-embedded kidney sections (3 μm) were examined using a Nikon Eclipse E600 (Tokyo, Japan) light microscope to assess the glomerulosclerotic index (GSI), as previously described [20].

### Tubulointerstitial fibrosis index

Masson’s trichrome-stained 3-μm paraffin-embedded kidney sections were examined using a Nikon Eclipse E600 light microscope to assess the TIFI, as previously described [26].

### Immunohistochemistry

Paraffin sections at 3 μm were incubated in 10% normal goat serum for 30 min at room temperature, followed by incubation overnight at 4°C with one of the following primary antibodies: MCP-1 (1:100, Santa Cruz), CD68 (1:100, Oncogene Sciences), and TGF-β (1:400, Santa Cruz). Negative controls were incubated in 10% normal goat serum only. Positive immunoreactivity was detected using the Envision Plus peroxidase method (Dako, Carpentaria, CA, USA), followed by counterstaining the slides with Mayer’s hematoxylin. The images were taken at × 400. The samples were quantitated by analyzing 25 randomly chosen visual fields per animal. The positive cell number was determined by counting the number of cortical positive cells (brown staining) in 25 different fields per animal from each group and expressed as the average percentage of brown staining in the field of view. Images were quantified using Image145 J software (National Institutes of Health http://rsbweb.nih.gov/ij/).

### Statistics

Statistical analysis between groups was performed using one-way ANOVA, with Student-Neuman-Keul’s *post hoc* tests. Significance was designated at *P* < 0.05.

## Results and discussion

### Effects of GH and sex on blood glucose and albuminuria

Blood glucose levels were elevated more than fourfold in all diabetic animals starting by week 1 (control, 100 ± 11 mg/dl; STZ, >400 mg/dl; STZ + GH, >400 mg/dl; *P* < 0.01, control vs STZ or STZ + GH) and remained elevated throughout the experimental period (data not shown). There was no additional effect of GH on blood glucose nor were there sex differences seen in blood glucose within treatment groups, with the limitation that all STZ groups were shown with blood glucose above 400 mg/dl.

Although the male rats were heavier than the females at the outset of the experiment, control rats of both sexes gained >15% body weight (BW) over the 8-week experiment (Table 1). STZ treatment caused the male rats to lose 29% of their BW over the experimental period. GH treatment had no additional effect on BW in the male rats. The females also lost BW after STZ treatment; however, the amount of BW loss (11%) was much less compared to the males.

**Table 1:** Effect of treatment on body weight, kidney weight, heart weight, and albuminuria of rats

	**Initial BW (g)**	**Final BW (g)**	**KW (g)**	**HW (g)**	**Albuminuria (mg/day)**
Female					
Control	244.5 ± 5.5	284.0 ± 9.0	1.52 ± 0.04	0.99 ± 0.11	2.9 ± 1.2
STZ	225.3 ± 1.8	201.0 ± 4.4^*^	2.20 ± 0.02^*^	0.94 ± 0.04	11.3 ± 2.7^*^
STZ + GH	227.4 ± 2.3	227.4 ± 18.7	2.36 ± 0.11^*^	0.95 ± 0.07	8.6 ± 1.8^*^
Male					
Control	369.7 ± 2.7	461.0 ± 14.0	2.68 ± 0.10	1.42 ± 0.02	3.1 ± 0.4
STZ	352.8 ± 8.1	251.8 ± 22.0^**^	2.75 ± 0.21	1.00 ± 0.08^**^	10.4 ± 0.8^**^
STZ + GH	353.0 ± 8.7	249.0 ± 25.6^**^	2.85 ± 0.19	1.13 ± 0.10^**^	16.1 ± 1.7^**,***^

*BW*, Body weight; *KW*, Kidney weight; *HW*, Heart weight. ^*^P > 0.05 vs sham female; ^**^P > 0.05 vs sham male; ^***^P > 0.05 vs STZ male by one-way ANOVA and Tukey’s post hoc tests. (*n* = 8 for each group).

Kidney weight (KW) was not affected by STZ treatment or GH supplementation in the male rats (Table 1). In contrast, KW increased by 1.4-fold in the females after STZ treatment, while GH supplementation had no additional effect.

STZ treatment reduced heart weight (HW) in the male rats and GH supplementation had no additional effect (Table 1). In comparison, STZ had no effect on HW in the females either in the presence or absence of GH supplementation.

Urine albumin excretion was elevated in both male (3.5-fold) and female (3.9-fold) STZ-treated rats compared with their same-sex controls (Table 1). GH supplementation increased the amount of albumin excreted in the diabetic male to 5.2-fold but had no additional effect on albuminuria in the diabetic female.

### Effects of GH and sex on glomerular and tubular damage

**Figure 1:**
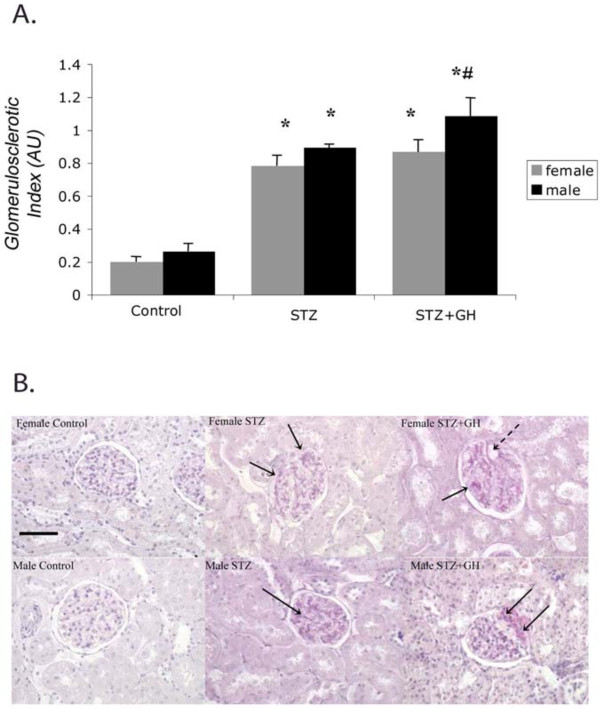
**Effect of sex on GSI in Control and diabetic rats treated with and without GH.** (**A**) STZ treatment significantly increased the GSI in both sexes, while GH treatment significantly increased GSI beyond STZ alone in males only.^*^P < 0.01 vs. control of the respective sex, ^#^*P* < 0.05 vs male STZ. (**B**) Representative pictures of periodic acid Schiff-stained sections. Diabetic kidneys were characterized by moderate glomerulosclerosis, as evidenced by mesangial expansion (*arrowheads*), and is seen more abundantly in male STZ + GH than STZ alone. Original magnification is × 400, and bar represents 100 μm.

STZ treatment was associated with modest glomerular injury in both sexes as evidenced by a fourfold increase in the GSI in both male and female diabetic rats (Figure 1A). GH supplementation magnified the effect of STZ on GSI by 30% in the male rats but had no additional effect in the females. Examination of the renal pathology indicates that the diabetes-induced renal damage was associated with modest mesangial expansion in both sexes (Figure 1B). These indicators of damage were more prevalent in the STZ + GH males.

**Figure 2:**
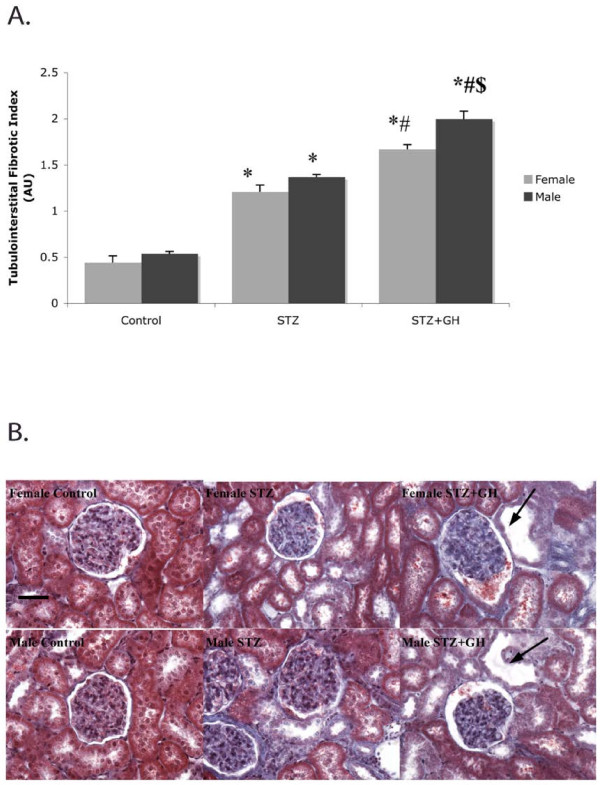
**Effect of sex on TIFI in control and diabetic rats treated with and without GH.** (**A**) STZ treatment significantly increases the TIFI in both sexes, and while GH treatment increased the TIFI in both sexes, the increase is greater in males than in females. ^*^P < 0.01 vs. control of the respective sex, ^#^*P* < 0.05 vs STZ of the respective sex, ^$^*P* < 0.05 vs female GH. (**B**) Representative pictures of Masson’s trichrome-stained sections. Diabetic kidneys were characterized by moderate tubule interstitial fibrosis, evidenced by the presence of extracellular matrix deposits (*blue staining*) and tubular dilation, and these changes (*blue staining* and *larger tubules*) are seen more abundantly in STZ + GH-treated animals, and most abundantly in STZ + GH-treated males. Original magnification is × 400, and bar represents 100 μm.

TIFI also increased after STZ (2.5-fold) treatment in both male and female rats. While GH supplementation further increased the TIFI in both sexes, the magnitude of this GH effect was greater in the male (20%) than in the female (5%) rats (Figure 2A). The tubular dilation and fibrosis observed in Masson’s trichrome-stained sections was magnified by the presence of GH in the diabetic animals (Figure 2B).

### Effect of sex and GH on renal markers of inflammation (MCP-1 and CD68)

**Figure 3:**
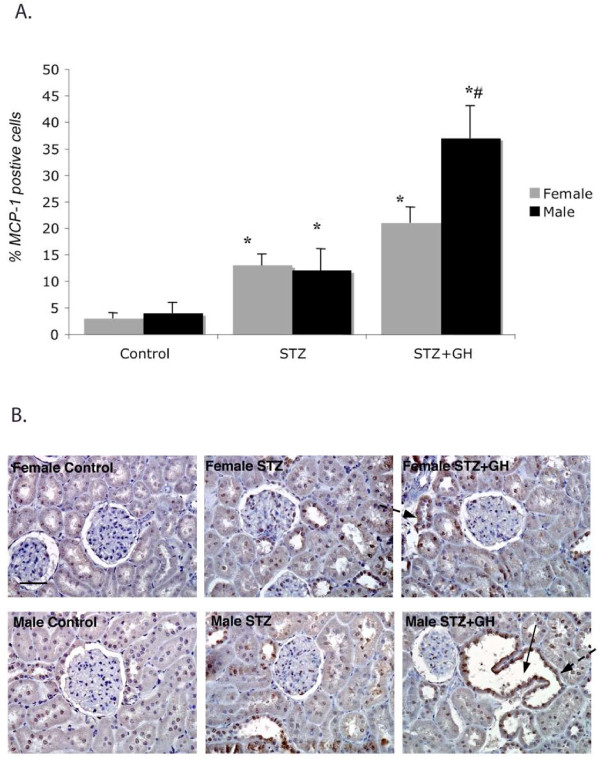
**Effects of STZ and GH treatment on renal MCP immunoreactivity.** (**A**) STZ increases the MCP-1 staining in kidneys from diabetic male and female rats; however, GH treatment increased MCP-1 only in kidneys from diabetic male rats. ^*^P < 0.01 vs control and ^#^*P* < 0.05 vs female. (**B**) Representative images show an increase in brown staining on dilated and injured tubules (*solid arrows* show dilated tubules, and *dashed arrows* show brown staining). Original magnification is × 400, and bar represents 100 μm.

MCP-1 is stimulated in response to pro-inflammatory cytokines (such as TGF-β) and stimulates immune cell infiltration of the tissue [24]. STZ treatment increased MCP-1-positive cells threefold in both male and female rats (Figure 3). While GH had no additional effect in female rats, there were 3.5 times more MCP-1+ cells in kidneys from STZ + GH male rats when compared to STZ alone (Figure 3A). Representative staining is shown in Figure 3B.

**Figure 4:**
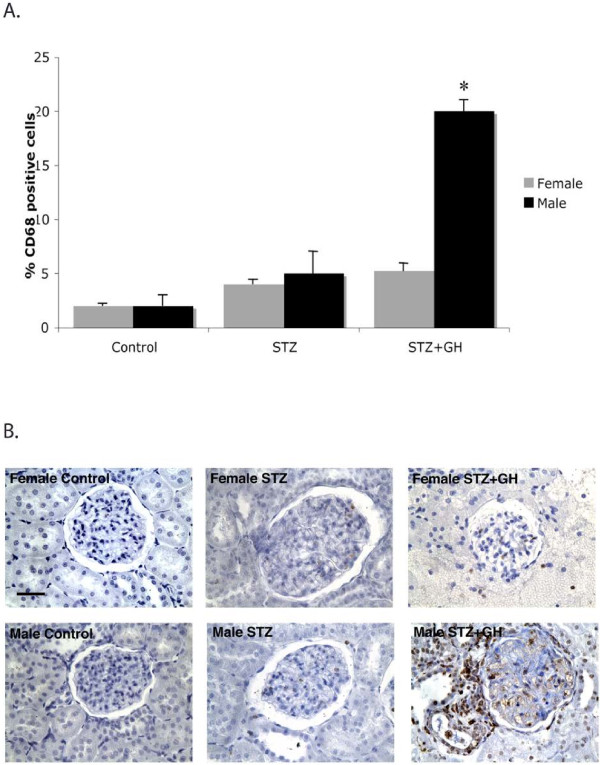
**Effects of STZ and GH treatment on renal CD68 immunoreactivity.** (**A**) No significant changes were observed in CD68 protein expression in kidneys from diabetic female rats. While CD68 staining was not increased in kidneys from STZ-only-treated rats, GH significantly increased CD68 staining in the glomeruli and tubules in diabetic male, but not female, rats. ^*^P < 0.01 compared to all other data (**B**) Representative images of CD68+ cells in kidney sections (*dark brown dots*). The intensity of staining is significantly increased in the STZ + GH-treated males. Original magnification is × 400, and bar represents 100 μm.

Representative staining of CD68+ cells, which indicates macrophage activation and tissue inflammation, is illustrated in Figure 4B. There was no evidence of CD68 staining in control animals of either sex. There were no significant changes in CD68 staining seen in kidneys from either male or female STZ-only-treated animals. However, in the presence of GH there was a significant fourfold increase in CD68+ cells (brown staining) in both the glomeruli and in surrounding tubulointerstitium in male kidneys, *but not in female kidneys* (Figure 4A,B). This finding further supports the concept that the male kidney is a target for GH-related damage and inflammation.

### Effect of sex and GH on renal TGF-β and MMP-2 and MMP-9

**Figure 5:**
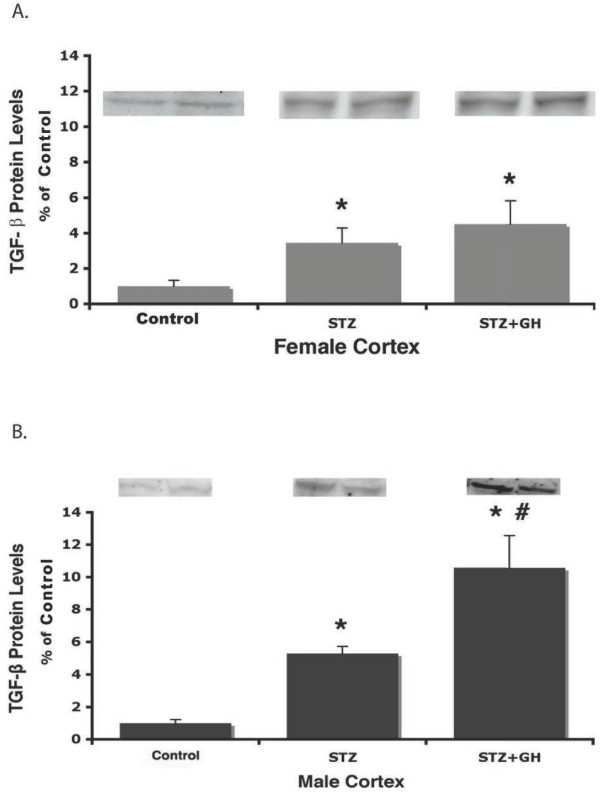
**Effect of sex on renal cortical TGF-β protein abundance in control and diabetic rats treated with and without GH.** (**A**) STZ and STZ + GH treatment increased female cortical TGF-β protein levels from control equally. ^*^P < 0.01 vs. female controls. (**B**) STZ treatment increased male TGF-B levels significantly, and STZ + GH significantly increased those levels. ^*^P < 0.01 vs. male controls, ^#^*P* < 0.05 vs. male STZ.

The effect of GH on renal TGF-β protein expression in diabetic kidneys is illustrated in Figure 5. STZ treatment increased TGF-β protein expression equally in both the male (Figure 5A) and female kidney cortexes (Figure 5B) (approximately fivefold greater than control, *P* < 0.05). However, while GH had no additional effect on TGF-β protein in diabetic female kidneys, there was a dramatic, GH-dependent, tenfold increase in TGF-β protein in male kidneys (*P* < 0.01).

**Figure 6:**
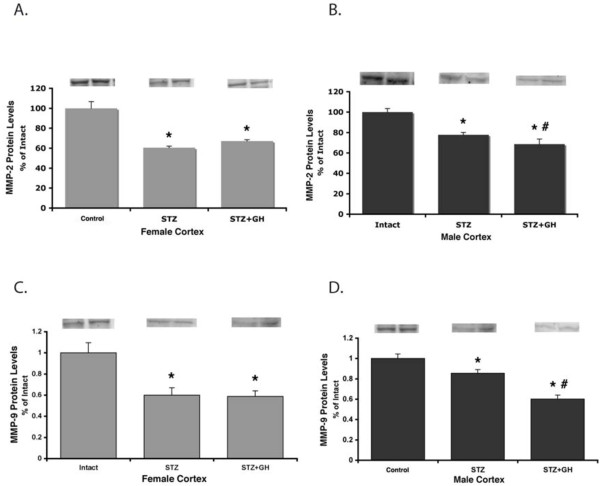
**Effect of sex on renal cortical MMP-2 and MMP-9 protein abundance in control and diabetic rats treated with and without GH.** (**A**) STZ and STZ + GH treatments significantly decrease MMP-2 levels in female mice compared to control. ^*^P < 0.01 vs. female controls. (**B**) STZ significantly decreases MMP-2 protein levels in male mice compared to control, and STZ + GH treatment is significantly lower than STZ alone. ^*^P < 0.01 vs. male control, ^#^*P* < 0.05 vs. male STZ. (**C**) STZ and STZ + GH treatments significantly decrease female MMP-9 levels than in controls. ^*^P < 0.01 vs. female controls. (**D**) STZ significantly decreases MMP-9 protein levels in male mice compared to control, and STZ + GH treatment is significantly lower than STZ alone. ^*^P < 0.01 vs. male control, ^#^*P* < 0.05 vs. male STZ.

There was a significant 20% decrease in both MMP-2 and MMP-9 protein expression in kidneys from male STZ-diabetic rats compared to values in nondiabetic controls. Interestingly, MMP-2 and MMP-9 protein expression was reduced by 40% in female STZ-treated animals compared to controls (Figure 6A–D). However, while GH further decreased MMP-2 and MMP-9 in the male kidneys, there was no further effect of GH in female STZ-treated kidneys (Figure 6B,D).

### Effect of GH on glomerular AT_1_R binding

Renal glomerular AT_1_R binding was tested. As previously reported [25], AT_1_R binding was significantly lower in the glomeruli from nondiabetic female animals compared with male animals (4,234 ± 178 cpm vs. 6,742 ± 210 cpm, *P* < 0.05). AT_1_R binding was not significantly changed in STZ-only-treated male and female glomeruli (3,934 ± 1,084 cpm vs. 5,037 ± 596 cpm). However, in both male and female STZ + GH-treated rats, AT_1_R binding significantly increased as compared to control and STZ-only-treated rats (8,462 ± 923 cpm vs. 9,562 ± 1,472 cpm, *P* < 0.05) (*graph not shown*).

## Conclusions

While previous studies have highlighted the issues of sex differences and GH effects in diabetic renal disease separately, the present work shows sex-related differences in response to GH in diabetic renal disease. The presence of GH appears to be necessary for the development of significant renal damage, but there are sex differences in the pattern of effects of GH on the diabetic kidney. Our findings have determined that GH has a greater overall effect in the male animals in proteins associated with renal damage (increased TGF-β, MCP-1 and CD-68, and lowered MMPs) and markers of pathology (increased GSI, TIFI, and albuminuria). GH was given at a fixed amount that has been shown to achieve levels similar to that observed in adult male rats (unpublished data). Because it was not adjusted for BW, the female rats received a greater proportional dosage, yet it did not exacerbate the renal damage, providing further evidence that the effect on the kidneys is specific to male animals. These findings extend previous reports and provide intriguing areas for future study. Furthermore, the fact that human diabetes is associated with overall elevated plasma GH [8,12] increases the relevance of the present work.

The STZ-treated rat is an excellent model for studying the role of GH in diabetes, since STZ treatment results in the suppression of pulsatile GH release and low plasma GH levels [11]: this low GH model can then be compared to STZ + GH replacement to identify GH-related changes in diabetes. This has allowed us to identify the differential patterns of response to GH in male and female diabetic animals. Consistent with previous reports, in both male and female animals, STZ treatment increases cortical TGF-β expression and decreases cortical MMP-2 and MMP-9 [27,28]. While GH increases AT_1_R binding in the diabetic female kidney, it does not increase other proteins associated with renal inflammation and damage (e.g., TGF-β, MCP-1, CD68, and MMPs) or exacerbate renal pathology. Our findings indicate that there are both AT_1_R-dependent and AT_1_R-independent actions of GH in the female diabetic kidney. Males showed a similar response to STZ, but all of the protein changes were amplified with GH and directly associated with exacerbation of renal morphologic damage. This differential GH action may be mediated by interactions involving testosterone and GH. Testosterone treatment alone has been shown to increase renal TGF-β mRNA and protein levels *in vivo*, which was followed by an increase in glomerulosclerosis as well as an increase in lymphocyte and macrophage infiltration [29]. These protein changes and resulting pathology were reversed with flutamide treatment (a nonsteroidal antiandrogen). In the present study, we did not see sex differences with STZ treatment alone, suggesting that both testosterone and GH are needed to uncover these sex differences.

Evidence suggests that a pro-inflammatory response contributes to the development of diabetic renal disease [30-32]. The infiltration of immune cells seen in many glomerular diseases could cause structural damage through the release of proteolytic enzymes and oxygen-free radicals, glomerular remodeling by the release of growth factors, or glomerular functional alternations through the release of pro-inflammatory cytokines [33,34]. Previous studies have shown that elevations in cytokines, such as TGF-β and IL-6, presage the development of the disease [30,35,36]. This pattern has also been found in the current study, and importantly, we found sex differences associated with this pro-inflammatory response. In strong support of differential GH-related mechanisms is the finding that GH has a dramatic effect on renal TGF-β in diabetic male animals, but not in female animals. This increase is directly correlated with exacerbated renal damage (increased GSI, TIFI, and albuminuria) in the male kidney. A previous study in STZ-diabetic mice reported that MCP-1 promotes renal damage [31]. In our study, elevations in MCP-1 were also associated with renal damage, but importantly, the stimulation of MCP-1 by GH is also affected by the sex of the animal. Interestingly, while MCP-1 is elevated to the same extent in the STZ-only-treated male and female rats, CD68+ cells (representing activated macrophages) are only elevated in the STZ + GH-treated male rat kidneys. This suggests that the 2-month period of untreated diabetes is sufficient to produce renal damage but that the exacerbation of the damage in the male may be due to the activation of the macrophages, whereas the pro-inflammatory damage observed in the STZ-only-treated animals may be independent of this effect.

The stimulation of TGF-β by GH has been reported by others [5]; however, sex differences are a novel finding and suggest that another mechanism is modulating the effect of GH on TGF-β in the female. Potentially, GH might use testosterone as a mediator for an increase in TGF-β; this might provide a layer of protection in the female against some of the early damage observed in the diabetic male. Indeed, a relationship has been suggested between E_2_ and TGF-β since E_2_ supplementation in (nondiabetic) TGF-β transgenic mice significantly reduces GS and TIF [37]. In our diabetic females, the presence of E_2_, albeit at a reduced level [26], may provide this protection against the actions of GH on TGF-β. Future studies in ovariectomized diabetic females with or without GH could illuminate this issue.

The concept that GH may provide another target for therapy in diabetes has been suggested [10] and is supported by many animal studies over the past 15 years [9,10,37]. Our data support the idea of this GH pharmacologic therapy for the treatment of diabetic kidney disease but suggest that it is especially relevant in males. Previous reports of GH antagonist treatment from the onset of diabetes found a normalization of diabetes-induced renal effects, including reduced renal hypertrophy, glomerular hypertrophy, and urine albumin content; however, these studies were *only* in male experimental animals [38]. It has also been shown that late intervention with a GH antagonist resulted in regression of some of diabetes-associated renal changes [9]. The present findings suggest that this potential therapy may be useful only in men.

In conclusion, sex differences exist in the mechanisms governing early, uncontrolled diabetes in the presence of GH. The male response to GH indicates significantly more pathology and damage than in females, suggesting evidence for differential sex-related pharmacologic treatments.

## Competing interests

The authors declare that they have no competing interests.

## Authors’ contributions

JW carried out all experimentation and data analysis. Funding was provided by SM, KS, AM, and CM. All authors provided support and expertise for the writing of this paper. All authors read and approved the final manuscript.
